# Relationship between gene responses and symptoms induced by *Rice grassy stunt virus*

**DOI:** 10.3389/fmicb.2013.00313

**Published:** 2013-10-18

**Authors:** Kouji Satoh, Kaori Yoneyama, Hiroaki Kondoh, Takumi Shimizu, Takahide Sasaya, Il-Ryong Choi, Koichi Yoneyama, Toshihiro Omura, Shoshi Kikuchi

**Affiliations:** ^1^Research Team for Vector-Borne Plant Pathogens, National Agricultural Research CenterTsukuba, Japan; ^2^Plant Genome Research Unit, Agrogenomics Research Center, National Institute of Agrobiological SciencesTsukuba, Japan; ^3^Weed Science Center, Utsunomiya UniversityUtsunomiya, Japan; ^4^Plant Breeding, Genetics, and Biotechnology Division, International Rice Research InstituteMetro Manila, Philippines

**Keywords:** *Rice grassy stunt virus*, plant hormone, stunting, tillering, transcriptome analysis

## Abstract

*Rice grassy stunt virus* (RGSV) is a serious threat to rice production in Southeast Asia. RGSV is a member of the genus *Tenuivirus*, and it induces leaf yellowing, stunting, and excess tillering on rice plants. Here we examined gene responses of rice to RGSV infection to gain insight into the gene responses which might be associated with the disease symptoms. The results indicated that (1) many genes related to cell wall synthesis and chlorophyll synthesis were predominantly suppressed by RGSV infection; (2) RGSV infection induced genes associated with tillering process; (3) RGSV activated genes involved in inactivation of gibberellic acid and indole-3-acetic acid; and (4) the genes for strigolactone signaling were suppressed by RGSV. These results suggest that these gene responses to RGSV infection account for the excess tillering specific to RGSV infection as well as other symptoms by RGSV, such as stunting and leaf chlorosis.

## Introduction

Grassy stunt disease of rice caused by *Rice grassy stunt virus* (RGSV) is one of the severe virus diseases of rice in several Southeast Asian countries (Shikata et al., 1980; Ramirez, 2008). RGSV is a member of the genus *Tenuivirus*, and is transmitted by brown planthopper (BPH, *Nilaparvata lugens*) and by two other *Nilaparvata* spp. (Hibino, 1996). RGSV has a thin filamentous-shaped virion, and the genome is composed of six ambisense single-stranded RNA segments (RNA1—6) containing 12 open reading frames (Ramirez, 2008). RGSV RNA 1, 2, 5, and 6 correspond to the four RNA segments of the type member of *Tenuivirus, Rice stripe virus* (RSV). RGSV RNA 3 and 4 are unique in this genus. The phylogenic relationship among tenuiviruses, including RGSV and RSV, indicates that RGSV forms a group distinct from the other tenuiviruses (Ramirez, 2008). Typical symptoms induced by RGSV infection are leaf yellowing (chlorosis), stunting, and excess tillering (branching) (Shikata et al., 1980). Chlorosis and stunting are also observed in plants infected with other tenuiviruses, whereas excess tillering is a symptom specific to RGSV infection.

Plant disease symptoms caused by virus infection are accompanied by changes in the expression of the genes involved in morphogenesis and development (Dardick, 2007; Lu et al., 2012; Pierce and Rey, 2013). Thus, common and specific symptoms might be associated with common and specific responses of morphogenesis- and development-related genes (Dardick, 2007). *Nicotiana benthamiana* infected with either *Plum pox virus* (PPV) or *Tomato ring spot virus* (ToRSV) showed leaf chlorosis, implying that chloroplast functions might have been impaired by infection with the viruses (Dardick, 2007). Transcriptome analysis of PPV- and ToRSV-infected plants showed the suppression of genes functioning in chloroplasts, however, the genes encoding ribosomal protein functioning in chloroplasts were suppressed only in ToRSV-infected plants (Dardick, 2007). These results indicate that similarity in disease symptoms may not result from the similarity in gene responses to virus infection, and that the gene responses associated with particular symptoms may depend on the virus species.

In this study, we analyzed the gene expression profile in rice plants infected with RGSV to gain insight into RGSV-induced gene responses associated with the symptoms. The results suggested that symptoms such as stunting and leaf chlorosis caused by RGSV infection were associated with the suppression of genes related to cell wall, hormone synthesis and chlorophyll synthesis while excess tillering specific to RGSV infection is associated with the suppression of strigolactone signaling and GA metabolism.

## Materials and methods

### Virus, insect vector, and plant samples

RGSV was maintained in rice plants (*Oryza sativa* cv. Nipponbare) in an air-conditioned greenhouse (25–30°C) (Shimizu et al., 2013). One hundred rice seeds were sown in a pot (250 mm in diameter and 100 mm in height) that had been filled with commercial soil mixture (Bonsol; Sumitomo Chemical, Japan). Twelve-day-old rice seedlings were exposed to ~300 viruliferous or virus-free BPH (for mock inoculation) in an inoculation chamber (340 mm wide by 260 mm deep by 340 mm high) for 1 day. Forty-two inoculated plants were transplanted and grown in a plastic container (53 cm wide by 35 cm deep by 10 cm high) in the greenhouse. At 28 days post-inoculation (DPI), when disease symptoms such as stunting and leaf yellowing became evident, two newly developed leaves from each plant which exhibited disease symptoms were harvested, frozen in liquid nitrogen, and stored at −80°C. Sample preparations were independently repeated three times for microarray experiments. Infection with RGSV in plants was evaluated by enzyme-linked immunosorbent assay using an antiserum against RGSV.

### Microarray experiment and data analysis

Total RNA was extracted from leaf samples pooled from five independent RGSV-infected or mock-inoculated plants by the RNeasy Maxi kit (Qiagen, UK). A microarray experiment involving complementary RNA synthesis, hybridization, array scanning, and image processing was performed as described previously (Satoh et al., 2010). A gene was declared “expressed” if the average signal intensity of the gene was higher than 64 in plants. A significantly differentially expressed gene (DEG) between RGSV-infected and mock-inoculated plants was defined as an expressed gene with (1) a signal intensity ratio between RGSV-infected and mock-inoculated samples greater than 1.5, and (2) significant changes in gene expression between two plants (*P* ≤ 0.05 by a paired *t*-test; permutation: all; false discovery rate correction: adjusted Bonferroni method). The gene expression profile of RGSV-infected plant (data series GSE 25217 available at NCBI GEO, Supplementary Material 1) was analyzed by a 4 × 44 K microarray system (platform number GPL7252 available at NCBI-GEO) (Edgar et al., 2002). All data are Minimum Information About a Microarray Experiment (MIAME) compliant.

### Semi-quantitative RT-PCR

Complementary DNA (cDNA) fragments for transcripts of selected rice genes were synthesized using 1.0 μg of total RNA with 50 ng/μL of random hexamer primers by SuperScript III reverse transcriptase (Invitrogen, USA). The resultant reaction mixtures containing cDNA were diluted four times. Four μL of diluted mixture was used for PCR. Primers for selected rice genes are shown in Supplementary Material 2. The cycling program was initial denaturation for 2 min at 95°C, followed by 30–40 cycles of 15 s at 95°C, 15 s at variable annealing temperatures, and 45 s at 68°C, with a final extension of 1 min at 68°C. Annealing temperature was adjusted depending on the Tm of designed primers, and was between 50 and 60°C.

### Quantification of strigolactone

RGSV-infected and mock-inoculated rice plants were prepared as described previously and grown for 6 weeks. Then, the plants (*n* = 20) were transferred to 1 L 1/2 Hoagland hydroponic culture medium without phosfate. After 2 days of acclimatization, culture media were collected and extracted three times with an equal volume of ethyl acetate containing 2′-epi-5-deoxystrigol-d6 (200 pg) as an internal standard. The ethyl acetate solutions were combined, washed with 0.2 M K_2_HPO_4_ (pH 8.3), dried over anhydrous MgSO_4_, and concentrated *in vacuo* to produce crude root exudate samples. Crude samples dissolved with 1 mL 90% (v/v) ethyl acetate/hexane were passed through Sep-pack silica cartridges. The eluents were concentrated *in vacuo* and the residues were taken up with a small volume of acetonitrile for liquid chromatography–mass spectrometry (LC-MS)/MS analyses. Quantification of 2′-epi-5-deoxystrigol was conducted by LC-MS/MS as described previously (Yoneyama et al., 2011).

## Results

### Disease symptoms caused by RGSV infection

**Figure 1:**
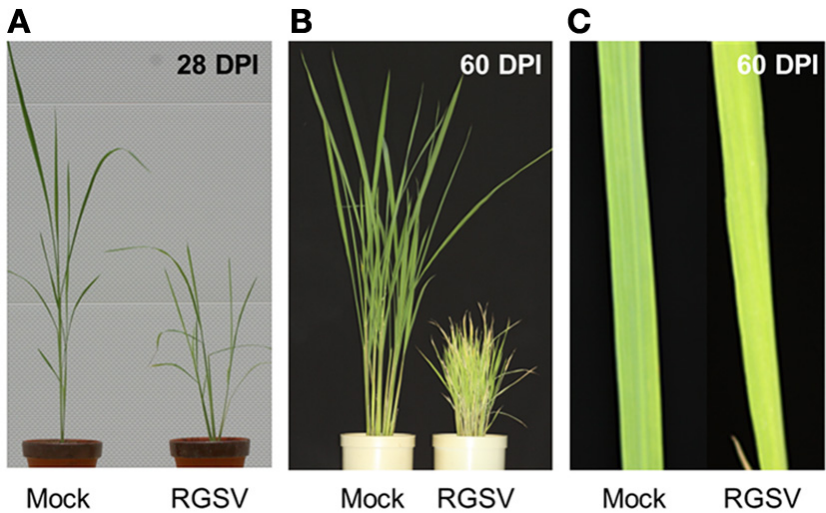
**Symptoms in rice caused by RGSV infection. (A)** Rice plants at 28 days and **(B)** 60 days after RSGV inoculation and mock inoculation. **(C)** Leaf yellowing of RGSV-infected plants at 60 days after inoculation.

Rice plants infected with RGSV showed disease symptoms such as excessive tillering, stunting, and leaf yellowing. The symptoms became more severe after 28 DPI (Figure 1A). Profuse tillering became more evident in the RGSV-infected plants at 60 DPI (Figure 1B). The RGSV-infected plant was much shorter than the mock-inoculated plants at 60 DPI (Figure 1B). Leaf yellowing by RGSV infection was also more severe at 60 DPI (Figures 1B,C) than at 28 DPI (Figure 1A).

### Transcriptome responses of rice to RGSV infection

Changes in gene expression caused by RGSV infection were examined by direct comparison between RGSV- and mock-inoculated rice plants at 28 DPI. The numbers of expressed genes and DEGs were 24,911 and 8203, respectively (Table 1, Supplementary Material 1). The expression patterns of some DEGs were confirmed by semi-quantitative RT-PCR (Supplementary Material 2).

**Table 1:** **Numbers of DEGs in gene ontology groups overrepresented or underrepresented in RGSV-infected plants**.

**Categories**	**Cene ontology ID^1^**	**Gene ontology term^a^**	**Number of expressed genes**	**Number of DEG^b^**	***P*-value^c^**	**Response of DEG**	
						**Induced**	**Suppressed**
Component	GO:0016020	Membrane	3193	**1188**	2.53E-05	671	517
	GO:0005618	Cell wall	1645	**688**	3.25E-10	377	311
	GO:0005623	Cell	233	**100**	7.88E-03	54	46
	GO:0005622	Intracellular	627	152	1.50E-04	103	49
	GO:0005840	Ribosome	301	43	1.73E-08	18	25
	GO:0005829	Cytosol	264	56	9.08E-04	33	23
Process	GO:0009719	Response to endogenous stimulus	2619	**1072**	9.56E-13	615	457
	GO:0007582	Physiological process	2105	**836**	5.78E-08	499	337
	GO:0007165	Signal transduction	2022	**785**	3.87E-06	485	300
	GO:0006950	Response to stress	1804	**694**	4.11E-05	411	283
	GO:0009628	Response to abiotic stimulus	1527	**676**	1.14E-14	404	272
	GO:0008150	Biological process	1388	**586**	1.63E-09	357	229
	GO:0009607	Response to biotic stimulus	1348	**585**	2.12E-11	321	264
	GO:0009058	Biosynthetic process	1222	**492**	7.94E-06	214	278
	GO:0006519	Amino acid and derivative metabolic process	803	**331**	4.23E-05	152	179
	GO:0019748	Secondary metabolic process	659	**317**	1.14E-11	133	184
	GO:0006629	Lipid metabolic process	655	**272**	1.26E-04	132	140
	GO:0006118	Electron transport	481	**210**	4.12E-05	98	112
	GO: 0005975	Carbohydrate metabolic process	473	**226**	1.82E-08	107	119
	GO: 0007275	Multicellular organismal development	463	**205**	2.09E-05	128	77
	GO:0030154	Cell differentiation	426	**183**	3.10E-04	120	63
	GO:0009908	Flower development	342	**164**	1.29E-06	92	72
	GO: 00096 53	Anatomical structure morphogenesis	204	**95**	6.87E-04	61	34
	GO:0016043	Cellular component organization and biogenesis	784	196	1.09E-04	117	79
	GO:0006412	Translation	516	83	2.60E-11	39	44
	GO:0006139	Nucleobase, nucleoside, nucleotide and nucleic acid metabolic process	453	95	9.20E-06	59	36
			24911	8204		3896	4307

a Based on Rice Genome Annotation Project database Osa1 (rice.plantbiology.msu.edu).

b Numbers in bold (or underlined) text indicate that the corresponding ontology groups are overrepresented (or underrepresented) based on a χ^2^-test between the actual ratio of DEG (the number of DEGs/the number of expressed genes belonging to the corresponding ontology group) and the expected ratio of DEG [the number of total DEGs (8204)/the number of the total expressed genes (24,911)].

c From the χ^2^-test.

We classified the DEGs in RGSV-infected plants according to gene ontology. For many ontology categories, the ratio of the number of DEGs to the number of expressed genes was significantly higher (*P* < 0.01 by a χ^2^ test) than the ratio of the total number of DEGs to the total number of expressed genes (Table 1). Based on categorization by cellular components, DEGs such as those categorized in “membrane” and “cell wall” were significantly overrepresented in the plants infected with RGSV, whereas the influence of RGSV infection on the expression of genes categorized in “ribosome” and “cytosol” appeared to be limited (Table 1). Based on categorization by cellular processes, DEGs involved in stress responses and secondary metabolism were overrepresented in the plants infected with RGSV (Table 1). For DEGs categorized in stress responses, the number of induced genes was significantly greater than that of suppressed genes, whereas the number of induced genes involved in secondary metabolism was not significantly different from that of suppressed genes. On the other hand, expression of genes involved in translation process and nucleic acid metabolism was less influenced by RGSV infection than genes in other categories (Table 1). These results indicated that genes involved in development of cell structures, secondary metabolisms, and stress responses were noticeably affected by RGSV infection, and might be associated with RGSV-induced symptoms.

#### Cell wall-related genes

The expression of genes related to cell wall components was affected by RGSV infection (Table 2). The expression of genes for cellulose synthase (-like) and arabinogalactan proteins associated with cell wall formation was predominantly suppressed by RGSV infection (Table 2). The genes encoding expansin proteins involved in cell elongation by loosening cell wall components (Choi et al., 2003) were also predominantly suppressed by RGSV infection (Table 2). These results indicate that RGSV infection suppressed both cell wall formation and cell elongation in rice.

**Table 2:** **Genes related to cell wall components and tillering in RGSV-infected plants that are up- or down-regulated**.

**Classification**	**Locus^a^**	**Log_2_ Ratio^b^**	**Response^c^**
Cellulose synthase	LOC_Os0lg54620	−0.70	D
	LOC_Os05g08370	−0.68	D
	LOC_Os07g2419	−1.03	D
	LOC_Os10g32980	−1.09	D
	LOC_Os12g29300	−0.77	D
Cellulose synthase-like family A	LOC_Os02g09930	−2.20	D
	LOC_Os02g51060	−0.60	D
	LOC_Os06g12460	−1.28	D
	LOC_Os08g33740	−2.03	D
Cellulose synthase-like family C	LOC_Os01g56130	−0.68	D
	LOC_Os03g56060	1.10	U
	LOC_Os08g15420	−0.84	D
Cellulose synthase-like family E	LOC_Os09g30120	1.13	U
	LOC_Os09g30130	−1.21	D
Cellulose synthase-like family F	LOC_Os07g36610	−0.89	D
	LOC_Os07g36690	−2.80	D
	LOC_Os07g36700	−1.99	D
	LOC_Os07g36740	−1.34	D
	LOC_Os08g06380	−0.92	D
Cellulose synthase-like family H	LOC_Os04g35030	1.35	U
Fasci din-like arabinogalactan protein	LOC_Os03g57460	−1.07	D
	LOC_Os07g06680	−0.73	D
	LOC_Os08g38270	−1.03	D
	LOC_Os05g07060	−1.76	D
	LOC_Os05g48900	−0.73	D
	LOC_Os08g39270	1.38	U
	LOC_Os05g38500	−0.99	D
	LOC_Os03g03600	−1.38	D
	LOC_Os08g23180	−1.02	D
	LOC_Os09g07350	−1.39	D
	LOC_Os09g30010	1.78	U
α-Expansin	LOC_Os01g14660	−2.34	D
	LOC_Os02g51040	−1.28	D
	LOC_Os03g60720	2.28	U
	LOC_Os04g15840	−1.12	D
	LOC_Os05g39990	−0.77	D
	LOC_Os06g41700	0.90	U
	LOC_Os10g30340	−1.24	D
β-Expansin	LOC_Os03g01270	−2.06	D
	LOC_Os 10g40710	−2.26	D
Expansin-like	LOC_Os03g04020	−0.90	D
	LOC_Os06g50960	−0.66	D
	LOC_Os10g39640	−1.36	D
Extensin	LOC_Os01g67390	−2.86	D
	LOC_Os04g32850	−1.66	D
	LOC_Os11g41120	1.73	U
OsNACs/Ostiin	LOC_Os04g38720	0.58	U
RCN1	LOC_Os03g17350	2. 47	U
SPL14	LOC_Os03g39890	−0.65	D

a Based on Rice Genome Annotation Project database Osa1 (rice.plantbiology.msu.edu).

b Log_2_-based differential expression ratio (signal intensity in RTSV-infected plant/signal intensity in mock-inoculated plant).

c U (D): Significantly induced (suppressed) by RGSV infection.

#### Genes associated with tillering

One of the major symptoms caused by RGSV infection is excessive tillering. The genes *RCN1* (*REDUCED CULM NUMBER 1*; Yasuno et al., 2009), *SPL14* (*SQUAMOSA PROMOTER BINDING PROTEIN-LIKE 14*; Miura et al., 2010), and *Ostill1* (*ORYZA SATIVA TILLERING 1*; Mao et al., 2007) were reported to promote shoot branching. RGSV infection induced the expression of *RCN1*and *Ostill1*, but suppressed that of *SPL14* (Table 2), suggesting that activation of *RCN1* and *Ostill1* alone may contribute to the development of excess tillers in RGSV-infected plants.

#### Genes functioning in chloroplast

**Figure 2:**
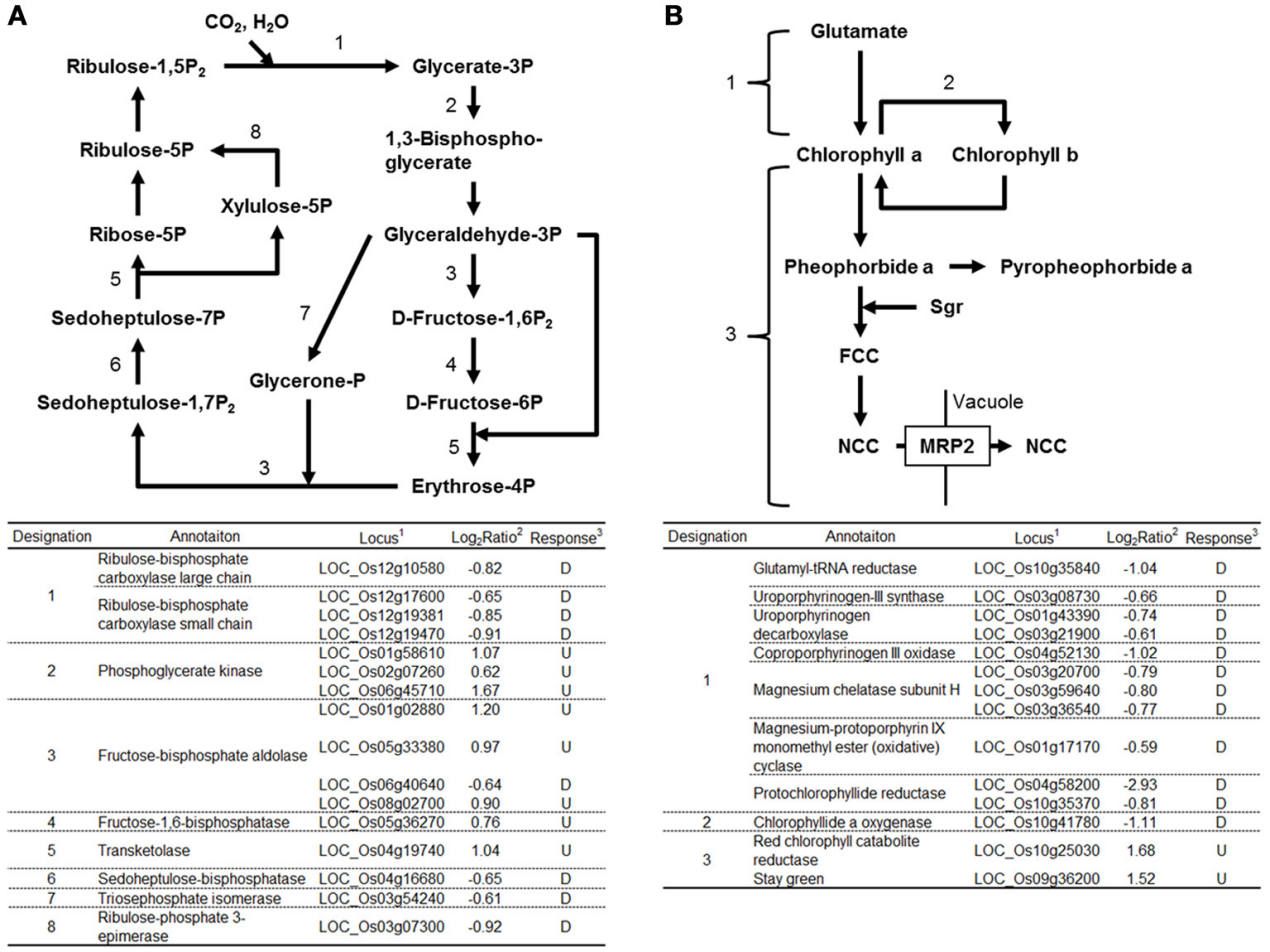
**Genes related to Calvin-Benson cycle and chlorophyll metabolisms in RGSV-infected plants that are up- or down-regulated**. Responses of genes for **(A)** Calvin-Benson cycle, and **(B)** chlorophyll metabolism. Enzymes catalyzing reactions are shown in the table below: ^1^based on Rice Genome Annotation Project database Osa1 (rice.plantbiology.msu.edu), ^2^log_2_-based differential expression ratio (signal intensity in RTSV-infected plant/signal intensity in mock-inoculated plant), and ^3^U (D): Significantly induced (suppressed) by RGSV infection.

Expression of many genes functioning in chloroplasts was affected by RGSV infection (Supplementary Material 1). Eight genes functioning in the Calvin-Benson cycle were induced by RGSV infection, whereas 8 genes including those encoding ribulose bisphosfate carboxylase (Rubisco) large and small subunits were suppressed (Figure 2A). Among 35 genes involved in the photosynthesis system whose expression was affected by RGSV, 34 were suppressed in RGSV-infected plants (Supplementary Material 1). The chlorophyll is a photoreceptor in the chloroplast. Among 14 genes for chlorophyll metabolism whose expression was affected by RGSV infection, 12 genes were suppressed and 2 genes were induced (Figure 2B). The suppressed genes were those involved in chlorophyll synthesis and conversion. The two induced genes were *Staygreen* (*Sgr*) and the gene encoding red chlorophyll catabolite reductase, both of which are associated with chlorophyll degradation (Park et al., 2007). Thus, it is likely that RGSV infection inhibits the carbon fixation and chlorophyll synthesis, and promotes degradation of chlorophyll.

### Expression of genes associated with plant hormone metabolism and signaling

We examined the expression patterns of genes involved in plant hormone biosynthesis and signaling processes. Rice genes for hormone biosynthesis and signaling and their orthologous genes in *Arabidopsis* are described in the Kyoto Encyclopedia of Genes and Genomes (KEGG) (Kanehisa et al., 2010).

#### Genes related to auxin biosynthesis and signaling

**Figure 3:**
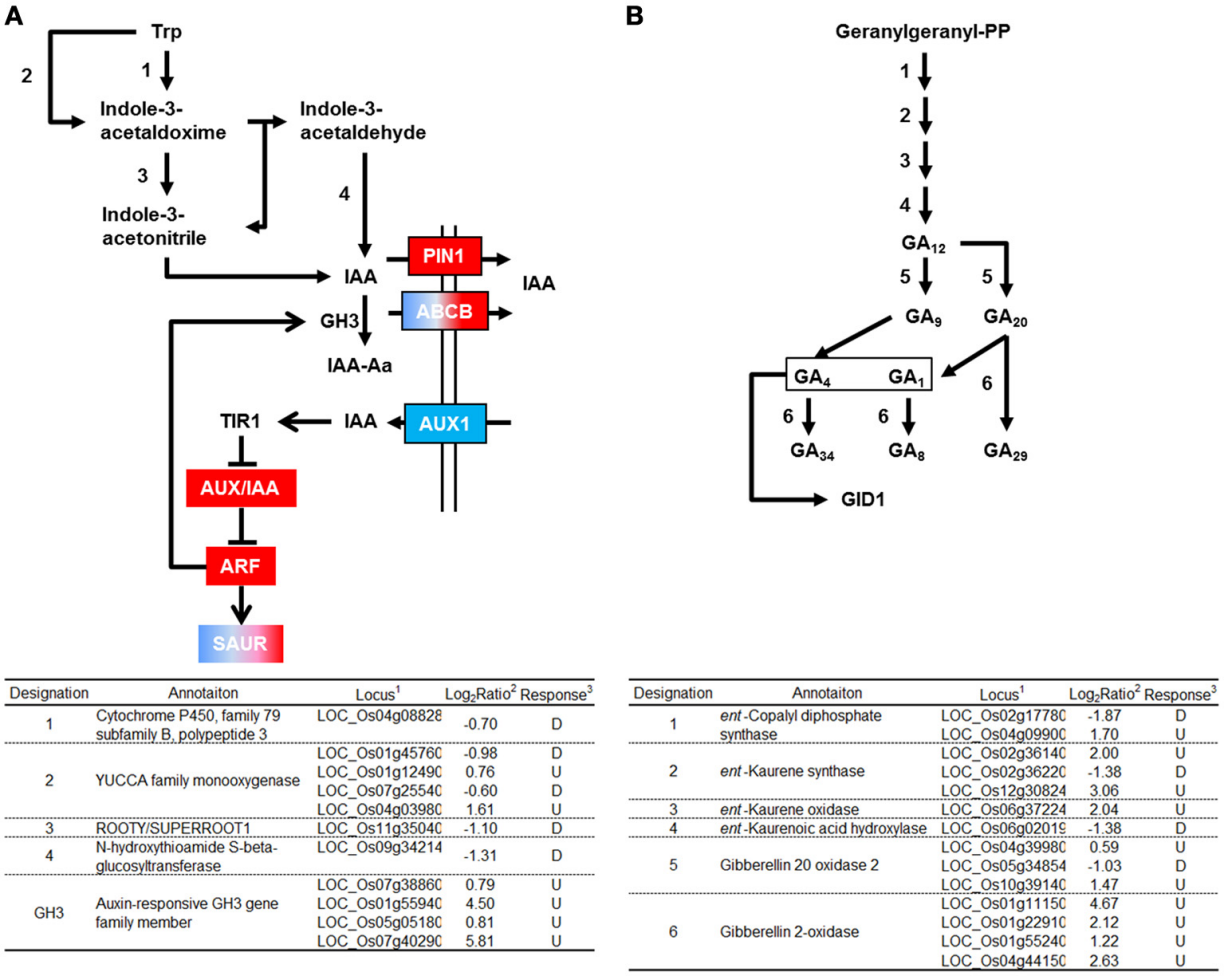
**Genes related to auxin and gibberellic acid biosynthesis and signaling in RGSV-infected plants that are up- or down-regulated**. Responses of genes for **(A)** auxin metabolisms and signaling, and **(B)** gibberellic acid (GA) metabolism. Enzymes catalyzing reactions are shown in the table below: ^1^based on Rice Genome Annotation Project database Osa1 (rice.plantbiology.msu.edu), ^2^log_2_-based differential expression ratio (signal intensity in RTSV-infected plant/signal intensity in mock-inoculated plant), and ^3^U (D): Significantly induced (suppressed) by RGSV infection. Boxes of red (or blue) indicate that the corresponding genes are predominantly induced (or suppressed). Boxes of red/blue indicate that the number of the corresponding genes induced was similar to that of suppressed genes. →: Reaction and translocation of substrate →: Positive signaling ⊣: Negative signaling.

GH3 converts IAA into an inactive form by conjugating IAA to amino acids (Domingo et al., 2009; Zhang et al., 2009). Four genes encoding GH3 were activated by RGSV infection (Figure 3A). RGSV infection also affected the expression of genes for auxin transporters such as *AUX1/LAX1, PIN1*, and *ABCB* (Figure 3A, Supplementary Material 1). RGSV suppressed *AUX1/LAX1* genes, but activated *OsPIN1* genes. RGSV infection induced the genes for the regulator of auxin signaling such as *AUX/IAA* and *ARF* (*AUXIN RESPONSE FACTOR*) (Lau et al., 2008) (Figure 3A). The expression of *SAUR* (*SMALL AUXIN-UP RNA*) genes involved in regulation of auxin synthesis and transport (Kant et al., 2009) was also affected by RGSV infection, and the number of induced *SAUR* genes was similar to that of suppressed genes (Figure 3A). These results suggest that RGSV infection might have inhibited the accumulation of auxin in rice, but activates IAA signaling process.

#### Genes related to gibberellic acid (GA) biosynthesis and signaling

Enzymes such as *ent*-kaurene synthase (KS), *ent*-kaurene oxidase (KO), and gibberellin 20-oxidase (GA20ox) catabolize the production of bioactive GA precursor (Yang and Hwa, 2008, and references therein). Five genes encoding these enzymes were induced by RGSV infection (Figure 3B). RGSV infection also activated the expression of four genes encoding gibberellin 2-oxidase (GA2ox), which degrades bioactive GA. The expression of genes for GA metabolism is regulated by Dwarf 62 (D62) and YABBY1 transcription factors (Dai et al., 2007; Li et al., 2010). The genes encoding D62 and YABBY1 were induced and suppressed by RGSV infection, respectively (Supplementary Material 1). On the other hand, the expression of genes encoding GID1, DELLA, and GID2, which are involved in GA signaling (Itoh et al., 2008), was not affected by RGSV infection (Supplementary Material 1). These results suggest that RGSV infection might have induced both GA biosynthesis and GA degradation.

#### Gene related to strigolactone (SL) biosynthesis and signaling

**Figure 4:**
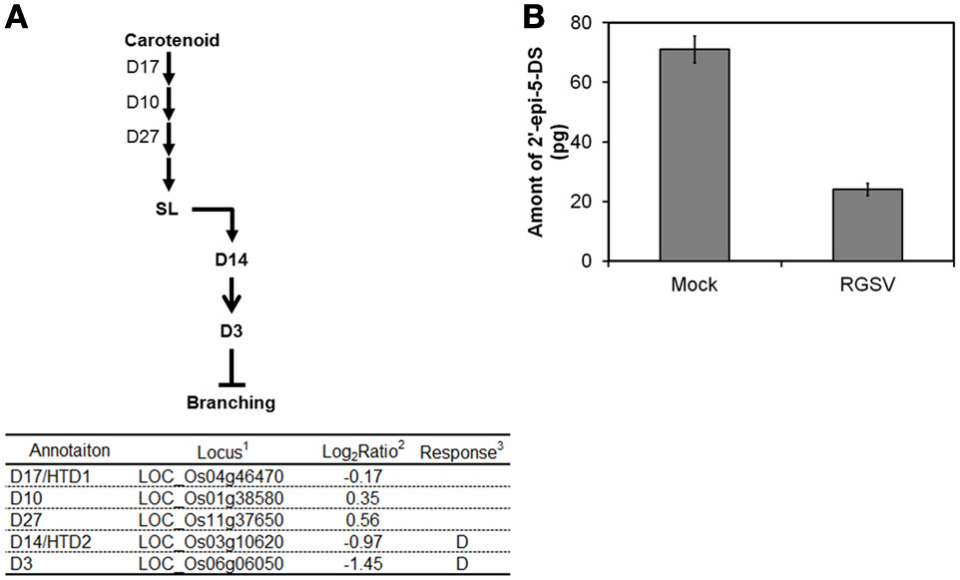
**Influence of RGSV infection on strigolactone biosynthesis and signaling**. Responses of genes for **(A)** strigolactone metabolisms and signaling. Enzymes catalyzing reactions are shown in the table below: ^1^Based on Rice Genome Annotation Project database Osa1 (rice.plantbiology.msu.edu), ^2^Log_2_-based differential expression ratio (signal intensity in RTSV-infected plant/signal intensity in mock-inoculated plant), ^3^D: Significantly suppressed by RGSV infection. **(B)** Strigolactone in root exudates from mock-inoculated and RGSV-infected plants. →: Reaction and translocation of substrate → : Positive signaling ⊣: Negative signaling.

SL was recently classified as a plant hormone. The expression of genes involved in SL biosynthesis such as those encoding Dwarf 17 (D17), D10, and D27 was not changed by RGSV infection (Figure 4A). We quantified 2′-epi-5-deoxystrigol, one of the major SLs n rice, in root exudates to examine whether endogenous SL level was changed by RGSV infection. Quantification of 2′-epi-5-deoxystrigol was made on plant tissue at 42 dpi instead of 28 dpi, because the symptoms induced by RGSV infection were clearly observed at this stage. The amounts of 2′-epi-5-deoxystrigol were low in both RGSV and mock plants (Figure 4B), but it seemed that RGSV infection suppressed the strigolactone synthesis. *HTD2* (*HIGH TILLERING DWARF 2*) and *D3* are related to SL signaling (Umehara et al., 2010; Yamaguchi and Kyozuka, 2010). The expression of the two genes was suppressed by RGSV infection. These results suggest that SL synthesis and signaling in rice might have been suppressed by RGSV infection.

## Discussion

We previously reported gene responses of rice to RSV and *Rice dwarf virus* (RDV) (Shimizu et al., 2007; Satoh et al., 2010, 2011). RSV is the type member of *Tenuivirus* and RDV belongs to the genus *Phytoreovirus*. Symptoms such as leaf stripe and stunting are common to plants infected with either virus. Symptoms caused by RSV infection are shown in Supplementary Material 3. The transcriptome analyses of plants infected with either virus commonly showed the predominant suppression of genes involved in cell wall synthesis, GA synthesis and chloroplast function, implying strong associations of such gene responses with leaf stripes and stunting. We expected that the symptoms of leaf yellowing, stunting and excessive tillering caused by RGSV are also associated with specific gene responses in RGSV-infected plants. In this study, we focused on the responses of genes involved in cell wall formation, chloroplast function, and hormone biosynthesis and signal transduction since expression of these groups of genes were also significantly affected in rice plants infected with RDV and RSV (Shimizu et al., 2007; Satoh et al., 2010, 2011).

### Gene expression responses associated with stunting and leaf yellowing in RGSV infected plants

RGSV infection induces stunting of rice plants. Many cellulose synthase (-like) genes were suppressed in RGSV-infected plants (Table 2). The suppression of many cellulose synthase (-like) genes was also seen in RSV-infected plants, which exhibits stunting symptom (Satoh et al., 2010). Expansins mediate loosening of cell wall. Suppression of *OsEXP4*, one of the rice expansin genes, resulted in stunted growth (Choi et al., 2003). The expression of many expansin (-like) genes were suppressed by RGSV infection. The suppression of many expansin genes was also observed in RSV-infected plants (Satoh et al., 2010). Therefore, the suppression of cellulose synthase and expansin genes is one of the common gene responses associated with stunting of rice infected with tenuiviruses.

The expression of the Rubisco genes was suppressed by RGSV infection (Figure 2A). The suppression of the Rubisco genes was also observed in RSV-infected plants (Satoh et al., 2010). RGSV-infected plants show leaf yellowing (Figure 1), implying the chlorophyll contents may have been decreased by RGSV infection. The appearance of leaf chlorosis in RSV-infected plants is different from that in RGSV-infected plants. RSV-infected plant showed white stripes on leaves (Satoh et al., 2010). The difference in leaf chlorosis pattern may have resulted from the difference in influence by RGSV and RSV on chlorophyll synthesis and degradation processes. In RGSV-infected plants, the expression of genes involved in chlorophyll synthesis was suppressed, and the suppression of those genes was also detected in RSV-infected plants. This suppression indicates that RGSV-infected plants contain low chlorophyll content, and have lower photosynthesis activity than healthy plants. These suggest that decreased biomass accumulation such as cell wall synthesis and carbon fixation activity may account for stunting of the virus-infected plants.

The difference in leaf chlorosis pattern may be related to chlorophyll degradation process (Figure 2B). The degradation of chlorophyll is regulated by *Sgr* gene (Park et al., 2007). Overexpression of *Sgr* resulted in yellowing leaves which is similar to the disease symptoms of RGSV-infected plants (Park et al., 2007). The expression of *Sgr* gene was induced by RGSV infection (Figure 2). Thus, the activation of *Sgr* by RGSV infection may play a role to induce the leaf yellowing symptom. In RSV-infected plants, the expression of genes involved in chlorophyll degradation was also activated, but the expression of *Sgr* was not significantly changed (Satoh et al., 2010). Therefore, the difference in the appearance of leaf chlorosis between the plant infected with RGSV and that infected with RSV may be related to the activation of *Sgr*.

Cell elongation is controlled by plant hormones such as GA and IAA. The expression of genes for GA biosynthesis such as those for KS, KO, and GA20ox, and those involved in GA inactivation such as the genes for GA2ox increased in RGSV-infected plants (Figure 3B), whereas the expression of these genes decreased in plants infected with RSV (Satoh et al., 2010). It was reported that rice plants overexpressing *GA2ox* contained lower bioactive GA, and were shorter than the wild type (Lo et al., 2008). The expression of *GA2ox* increased in stunted rice plants after infection with RDV (Satoh et al., 2011). These observations, in total, suggest that there are multiple ways to reduce the bioactive GA content and produce stunted plants, and that for RGSV an induction of *GA2ox* may aid in producing the stunted plants. On the other hand, many genes for both GA biosynthesis and degradation were suppressed in plants infected with RSV, which may have caused a reduction in GA content. Sakamoto et al. (2004) reported that a GA-deficient mutant was stunted. Therefore, the stunting symptom of RSV-infected plants might be related to GA deficiency. These observations indicate that RGSV- and RSV-infected plants may contain low bioactive GA, though the pathways to the reduction may be different between the plant infected with RGSV and that infected with RSV.

RGSV infection suppressed the genes for IAA biosynthesis, and induced the genes for IAA inactivation (Figure 3A). This implies that IAA content decreases in plants infected with RGSV. In addition, RGSV infection increased the expression of *GH3* genes (Figure 3A). It is known that the activation of *GH3* genes inhibits plant growth by suppressing genes related to auxin biosynthesis and signaling, and those for expansin (Ding et al., 2008; Domingo et al., 2009). The activation of *GH3* genes was also observed in RSV-infected plants (Satoh et al., 2010). These observations suggest that stunting of plants infected with rice tenuiviruses is associated with a reduction in auxin content.

Overexpression of a SAUR gene resulted in various morphological changes in rice plants (Kant et al., 2009). The expression of a SAUR gene was conspicuously induced in *Arabidopsis thaliana* by *Beet curly top virus* (BCTV), and the induction of the SAUR gene was correlated with symptom development and tissue-specific accumulation of BCTV (Park et al., 2004). The expression of many SAUR genes was found to be regulated in the plants infected with RGSV (Supplementary Material 1, Figure 3A), RDV (Satoh et al., 2011), and RSV (Satoh et al., 2010). Among the regulated SAUR genes, *OsSAUR13, OsSAUR26*, and *OsSAUR33* were commonly activated by RGSV and RDV, whereas *OsSAUR8, OsSAUR30, OsSAUR44, OsSAUR46, OsSAUR53, OsSAUR57*, and *OsSAUR58* were commonly suppressed by RGSV, RDV and RSV. Consequences of the regulated expression of various SAUR genes in the RGSV-infected plant are not clear, but the SAUR genes commonly regulated by RGSV, RDV, and RSV may be associated with stunted growth of plants infected with these viruses.

### Gene associated with excess tillering by RGSV infection

Excess tillering (shoot branching) is RGSV-specific symptoms. Shoot branching is controlled by plant hormones (Dill and Sun, 2001; Ferguson and Beveridge, 2009; Shimizu-Sato et al., 2009). Auxin and SL inhibit shoot branching, whereas cytokinin promotes it. In RGSV-infected plants, genes related to IAA biosynthesis were suppressed and those related to IAA degradation were induced (Figure 3A), implying that the IAA content was decreased by RGSV infection. The suppression of genes involved in IAA synthesis genes and the induction of genes involved in IAA degradation genes were also observed in RSV-infected plants which do not show excess tillers. These indicate that the reduction in auxin content is not directly associated with the excess tillering of RGSV-infected plants. A rice plant overexpressing the *GH3-8* displayed phenotypes of dwarfism and excess tillering (Ding et al., 2008) which are similar to the symptoms of RGSV-infected plants. However, the activation of *GH3-8* was also observed in RSV-infected plants. Therefore, the induction of the *GH3* genes in RGSV-infected plants may not be related to excess tillering.

Rice genes *D10, D17/HTD1*, and *D27* are involved in SL biosynthesis, and *D3* and *D14/HTD2* are involved in SL signaling (Arite et al., 2009; Lin et al., 2009; Yamaguchi and Kyozuka, 2010). Rice mutants for these genes have more tillers than the wild type. The expression of *D3* and *D14*/*HTD2* was suppressed in rice plants infected with RGSV (Figure 4), implying that the SL signaling process is repressed by RGSV infection. In addition, endogenous SL was reduced by RGSV infection (Figure 4). The expression of *D17/HTD1* was also suppressed in RSV-infected plants. Thus, SL biosynthesis might have been suppressed by RSV infection. These results suggest that excessive tillering because of RGSV infection may be related to the repression of SL signaling, but not to the decrease in SL content in RGSV-infected plants.

A reduction in active GA also promotes the formation of axillary buds (Curtis et al., 2005; Lo et al., 2008). *GA2ox* is one of the key genes for GA degradation to produce inactive GA (Curtis et al., 2005; Lo et al., 2008). Overexpression of *GA2ox* in rice resulted in development of excess tillers, a decrease in bioactive GA, and an increase in inactive GA (Lo et al., 2008). RGSV infection induced *GA2ox* (Figure 3B). Thus, it is likely that RGSV infection decreases bioactive GA content and increase inactive GA content in rice plants. On the other hand, genes involved in biosynthesis and degradation of bioactive GA were suppressed by RSV infection (Satoh et al., 2010), indicating that GA content may have been decreased by RSV infection. Plants deficient in GA display stunted growth and reduced tillers (Margis-Pinheiro et al., 2005), which are similar to the symptoms caused by RSV. These observations suggest that excessive tillering because of RGSV infection is related to the accumulation of inactive GA, but not to the lack of bioactive GA.

Overall, excessive tillers induced specifically by RGSV infection might be a consequence from the additive actions of SL, GA, and the genes regulating shoot branching, such as *RCN1*and *Ostill1*. Examination for possible interactions between RGSV proteins and host proteins, and phenotypes of transgenic plants with RGSV genes may help to elucidate the molecular mechanism of excessive tillering caused by RGSV infection. Moreover, there could be modifications in gene expression that occur earlier than 28 dpi, and further studies will determine how such modifications influence the symptoms observed at 28 dpi.

### Conflict of interest statement

The authors declare that the research was conducted in the absence of any commercial or financial relationships that could be construed as a potential conflict of interest.

## Supplementary material

The Supplementary Materials for this article can be found online at: http://www.frontiersin.org/journal/10.3389/fmicb.2013.00313/abstract

Supplementary Material 1 **List of expressed genes and differentially expressed genes in RGSV-infected plants**.

Supplementary Material 2 **Examination of expression of selected genes by RT-PCR**. The numbers below DNA band images are average log_2_ (signal intensity of microarray data) for the corresponding genes in mock-inoculated or RGSV-infected plants (Supplementary Material 1).

Supplementary Material 3 **Symptoms in rice caused by RSV infection**. Rice plants at 60 days after RSV inoculation and mock inoculation.
