# Supplementary figures and images for: Relationship between gene responses and symptoms induced by Rice grassy stunt virus
Source: Front Microbiol. 2013 Oct 18;4:313. doi: 10.3389/fmicb.2013.00313 (PMC3798811; doi:10.3389/fmicb.2013.00313)

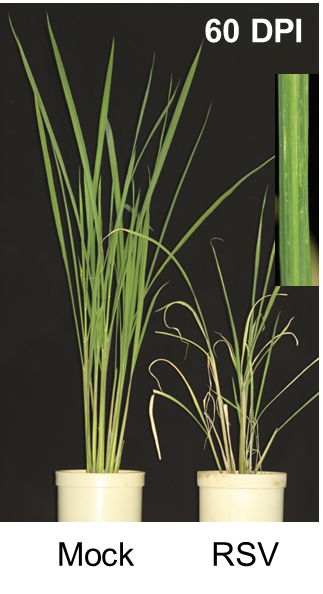

Supplement: Supplementary Material 1 — List of expressed genes and differentially expressed genes in RGSV-infected plants. [file DataSheet1.ZIP › Supplementray material 3.tif]

## Slide 1
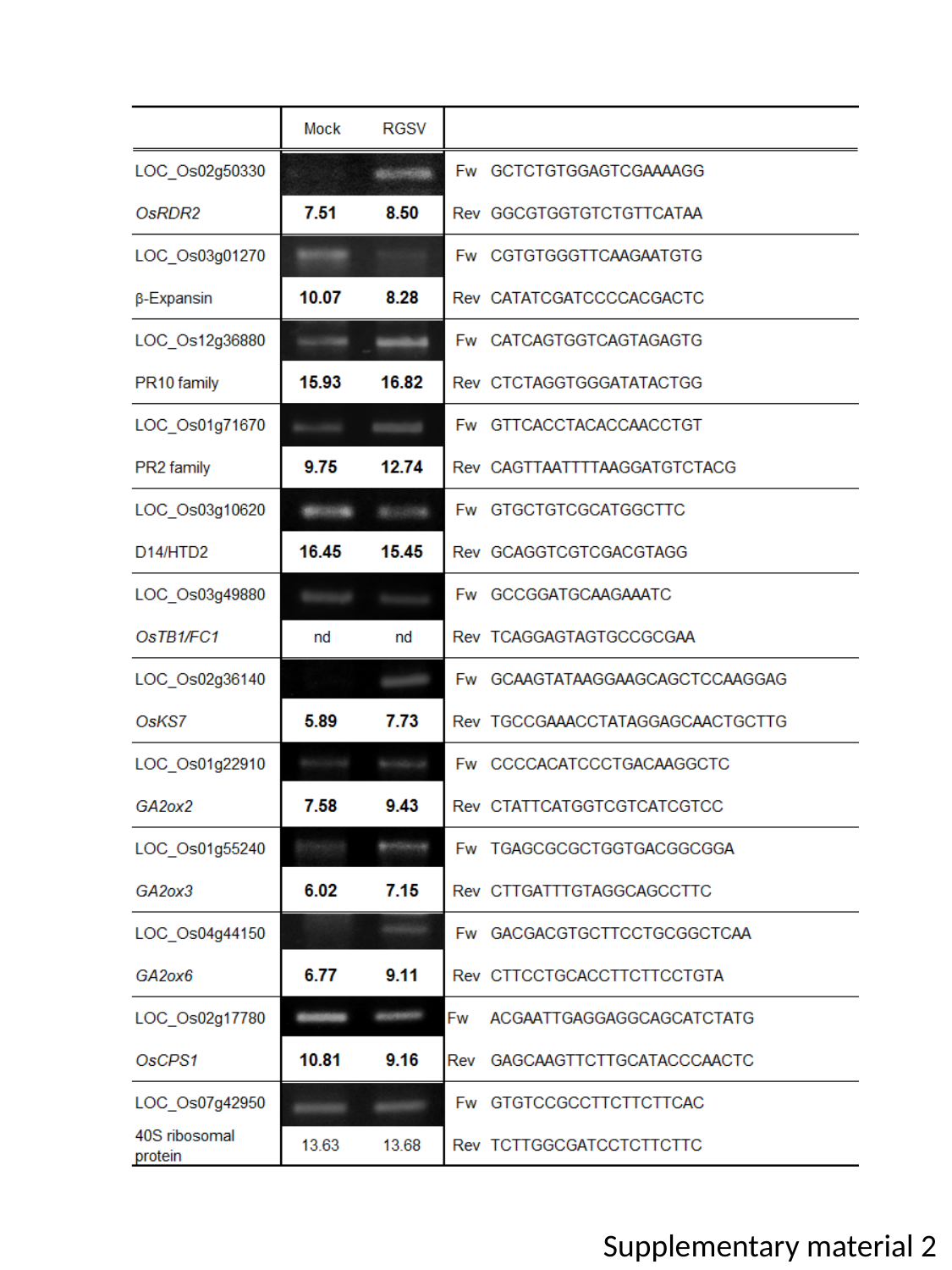

Supplementary material 2

Supplement: Supplementary Material 1 — List of expressed genes and differentially expressed genes in RGSV-infected plants. [file DataSheet1.ZIP › (20130915)Supplementary material2.pptx]
